# Evaluation of Fibroblasts Adhesion and Proliferation on Alginate-Gelatin Crosslinked Hydrogel

**DOI:** 10.1371/journal.pone.0107952

**Published:** 2014-09-30

**Authors:** Bapi Sarker, Raminder Singh, Raquel Silva, Judith A. Roether, Joachim Kaschta, Rainer Detsch, Dirk W. Schubert, Iwona Cicha, Aldo R. Boccaccini

**Affiliations:** 1 Institute of Biomaterials, Department of Materials Science and Engineering, University of Erlangen-Nuremberg, Erlangen, Germany; 2 Department of Cardiology and Angiology, University Hospital Erlangen, Erlangen, Germany; 3 Institute of Polymer Materials, Department of Materials Science and Engineering, University of Erlangen-Nuremberg, Erlangen, Germany; 4 Cardiovascular Nanomedicine Unit, Section of Experimental Oncology and Nanomedicine, ENT Department, University Hospital, Erlangen, Germany; Institute for Frontier Medical Sciences, Kyoto University, Japan

## Abstract

Due to the relatively poor cell-material interaction of alginate hydrogel, alginate-gelatin crosslinked (ADA-GEL) hydrogel was synthesized through covalent crosslinking of alginate di-aldehyde (ADA) with gelatin that supported cell attachment, spreading and proliferation. This study highlights the evaluation of the physico-chemical properties of synthesized ADA-GEL hydrogels of different compositions compared to alginate in the form of films. Moreover, *in vitro* cell-material interaction on ADA-GEL hydrogels of different compositions compared to alginate was investigated by using normal human dermal fibroblasts. Viability, attachment, spreading and proliferation of fibroblasts were significantly increased on ADA-GEL hydrogels compared to alginate. Moreover, *in vitro* cytocompatibility of ADA-GEL hydrogels was found to be increased with increasing gelatin content. These findings indicate that ADA-GEL hydrogel is a promising material for the biomedical applications in tissue-engineering and regeneration.

## Introduction

The insufficient availability of tissue donors and issues related to chronic immunosuppression are the major challenges for organ grafting. Although the last decade has brought about considerable progress in the field of tissue engineering and regeneration, transplantation is still the method of choice to replace damaged organs or tissues. Bioengineered organs can constitute a good solution to these problems due to their better availability, decreased risk of graft-*versus* host disease and thus, reduced rehabilitation time. Highly biocompatible materials are the obvious choice for the development of bioengineered organs. Currently, various materials of natural and synthetic origin are used for experimental work in the field of tissue engineering. The cell-material interaction represents the critical phenomenon for the evaluation of biocompatibility of a material for tissue engineering applications, and is reflected by the effect of the biomaterial properties on cell attachment, proliferation, spreading as well as cell differentiation [1]. Physico-chemical surface properties of the material, such as microstructure, topography, wettability, presence of functional groups, and stiffness have strong influence on the cell-material interaction [2]–[5]. Accordingly, it has been demonstrated that cell-material interaction can be improved by physico-chemical modifications of biomaterials, e.g. by plasma treatment, laser nano-patterning, and functionalization with chemical functional groups like amine, hydroxyl, and carboxyl groups [6]–[10]. Among the commonly used modifications, immobilization of biomolecules, such as cell adhesive peptides with the RGD sequence (Arg-Gly-Asp) has been widely used [11]–[14]. In our current study on soft matrices for cytocompatibility, alginate is being used as base biomaterial because of its biocompatibility and rapid ionic gelation property with divalent cation making it useful for biofabrication strategies [15], [16]. However, pure alginate as a biomaterial has a number of drawbacks, for example it does not effectively promote cell adhesion and possesses relatively slow and uncontrolled degradation kinetics in physiological conditions [17]–[19]. To overcome these limitations, we synthesized alginate-gelatin crosslinked (ADA-GEL) hydrogel by covalent crosslinking of oxidized alginate and gelatin. In the last few years, covalently crosslinked alginate-gelatin hydrogel draws the attention as a potential material in the field of tissue engineering. In the most of the studies, borax has been used for synthesizing covalently crosslinked alginate-gelatin hydrogel, which reduces the gelation time because of high pH [20]–[22]. However, short gelation time reduces the processing and handling time of hydrogel that abates its potential application for biofabrication. In our study, we used phosphate buffered saline (PBS) for synthesizing ADA-GEL hydrogel to optimize the gelation time, which is described in detail elsewhere [23]. Moreover, we have used low concentrations of ADA and gelatin compared to the previous studies, with different compositions to synthesize the crosslinked hydrogels for the future application in biofabrication process of tissue engineering. We hypothesized that this form of hydrogel could solve the two above mentioned major limitations of alginate, because oxidized alginate possesses comparatively high degradability [24] and gelatin possesses the RGD sequence of collagen which promotes cell adhesion [25], [26]. Moreover, we have focused on comparative cytocompatibility of ADA-GEL hydrogels of different compositions using primary human dermal fibroblast cells. Fibroblasts are the most abundant cells in various tissues and play an important role in wound healing, angiogenesis and tissue regeneration [27]–[30]. Fibroblasts secrete various growth and angiogenic factors, i.e. fibronectin, transforming growth factor (TGF-β1), basic fibroblast growth factor (bFGF), collagen I and III, connective tissue growth factor etc., and this feature makes them the key players in the control of the extracellular environment, as well as in the regulation of the neighboring cell behavior and their response to the environment [27], [31]–[33]. In the present study, to elucidate the influence of different compositions of the hydrogels on fibroblast cells, cell growth was observed over 7 days. Using biochemical and microscopic assays, we have addressed various questions related to cell growth and spreading on the hydrogels. In line with previous studies, it was found that different compositions and physico-chemical properties of the hydrogels effect cell behavior, morphology and functions. These results are discussed in the context of cell behavior with respect to different hydrogel compositions for the purpose of tissue regeneration.

## Materials and Methods

### Materials

Sodium alginate (sodium salt of alginic acid from brown algae, suitable for immobilization of micro-organisms, guluronic acid content 65–70%), and gelatin (Bloom 300, Type A, porcine skin, suitable for cell culture) were obtained from Sigma-Aldrich, Germany. Ethanol, ethylene glycol, sodium metaperiodate and calcium chloride di-hydrate (CaCl_2_.2H_2_O) were purchased from VWR International, Germany. Silver nitrate was from Alfa Aesar, USA.

### Synthesis of Alginate-Gelatin Crosslinked Hydrogels

Alginate-gelatin crosslinked (ADA-GEL) hydrogel was synthesized by covalent crosslinking of alginate di-aldehyde (ADA) and gelatin, as described in detail elsewhere [23]. Briefly, ADA was synthesized by controlled oxidation of sodium alginate in equal volume of ethanol-water mixture. 5 g of alginate were dispersed in 25 ml ethanol and mixed with 25 ml aqueous solution of sodium metaperiodate (7.5 mmol). The suspension was continuously stirred in dark condition at room temperature. The reaction was quenched after 6 hours by adding 5 ml of ethylene glycol (relative density 1.115) under continuous stirring for 30 minutes. The resultant suspension was dialyzed against ultrapure water (Direct-Q, Merck Millipore, Germany) using a dialysis membrane (MWCO: 6000–8000 Da, Spectrum Lab, USA) for 7 days with several changes of water until the dialysate was periodate free. The absence of periodate was checked by adding a 0.5 ml aliquot of the dialysate to 0.5 ml of a 1% (w/v) solution of silver nitrate and ensuring the absence of any precipitate. The ADA solution was then frozen and lyophilized. Gelatin solution (5% w/v, in ultrapure water) was added slowly into the solution of ADA (5% w/v, in PBS) under continuous stirring to facilitate crosslinking between ADA and gelatin. The weight ratios of ADA and gelatin in the final hydrogels were 70/30, 60/40, 50/50, 40/60 and 30/70 and their compositions are shown in Table 1.

**Table 1:** Labels used for different samples as a function of their composition.

Weight ratios (%)		Final Concentration (w/v%)		Labels for composition
ADA	Gelatin	ADA	Gelatin	
70	30	3.5	1.5	ADA70-GEL30
60	40	3	2	ADA60-GEL40
50	50	2.5	2.5	ADA50-GEL50
40	60	2	3	ADA40-GEL60
30	70	1.5	3.5	ADA30-GEL70

### Molar Mass Measurement

The molar mass of alginate and synthesized ADA were determined using the viscosity method [34]–[36]. Sodium alginate was dissolved in 0.1 M NaCl solution to get the final concentrations 0.05, 0.1 and 0.15% (w/v). For ADA, the concentrations of solutions were 0.1, 0.2, 0.3 and 0.4% (w/v). The experiment was carried out at 25°C with Ubbelohde viscometer (Schott-Geräte GmbH, Germany). The viscosity average molar mass (*M_η_*) of sodium alginate and ADA were calculated from its measured intrinsic viscosity [*η*] according to the following Mark-Houwink equation by adapting *a* and *K* values from Smidsrød [36]

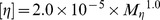
assuming that it holds for alginate and synthesized ADA.

### Preparation of Films

Alginate was dissolved in PBS to obtain 2.5% (w/v) solution and sterilized by filtration through 0.45 µm filter (Carl Roth GmbH + Co. KG, Germany). ADA and gelatin solution were also sterilized by filtration through 0.45 µm and 0.22 µm filter, respectively prior to synthesizing ADA-GEL hydrogels. ADA-GEL hydrogels of different compositions were prepared as described before. Alginate and ADA-GEL hydrogels were casted in sterile Petri dishes separately, followed by the addition of calcium chloride solution (0.1 M) and incubation for 15 minutes to allow ionic gelation. The hydrogels were then washed with serum-free Dulbecco's modified Eagle's medium (DMEM) (Gibco, Germany) and cut by punching to produce the films of desired size and shape (circular, diameter 13.5 mm and thickness 1.5 mm). The whole process was done under sterile conditions in a laminar flow hood (SCANLAF MARS Bio-safety cabinet class 2, LaboGene, Denmark).

### Water Uptake Behavior

Water uptake studies of films prepared from different hydrogels were performed in DMEM supplemented with 10% (v/v) fetal calf serum (FCS) (Sigma-Aldrich, Germany) and 1% (v/v) antibiotic-antimycotic (Gibco, Germany) at 37°C. The fabricated films of different hydrogels were dried with a critical point dryer (Leica EM CPD300, Germany). The weight of the critical point dried films (*W_d_*) was recorded prior to the immersion in DMEM. At different time points, the DMEM was removed and the surface of the films was blotted with blotting paper to remove adherent DMEM, following which the films were weighed (*W_s_*). Fresh culture medium was then added to the films. The water uptake was calculated using the following equation: 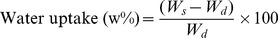



### *In Vitro* Swelling and Degradation Study

The weighed (*W_i_*) as fabricated films of alginate and ADA-GEL of all compositions were incubated in 3 ml DMEM supplemented with 10% (v/v) FCS and 1% (v/v) antibiotic-antimycotic at 37°C with a controlled atmosphere of 5% CO_2_ and 95% relative humidity. The medium was changed every two days. After specific time intervals, surface culture medium was removed from the samples and weighed (*W_t_*). The fresh culture medium was then added to the samples. The swelling and degradation of the films were calculated according to the following equation: 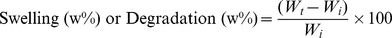
where, positive values are considered as swelling (w%) and negative values are considered as degradation (w%).

### Gelatin Release Behavior

Weighed films (125 mg) prepared from ADA-GEL hydrogels, were immersed in 2 ml of L-glutamine, phenol red and serum free DMEM and incubated at 37°C. At selected time points, the medium was removed and collected for gelatin release analysis, and fresh DMEM (2 ml) was added to the films. The gelatin concentration in the released medium was determined by colorimetric protein assay using the Lowry method [37], [38], with bovine serum albumin (BSA) as a standard. The absorbance of each solution at 750 nm was measured using a UV-Vis spectrophotometer (Specord 40, Analytik Jena, Germany). The release (%) of gelatin from the films was calculated as follows: 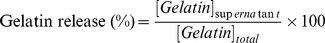
where, [*Gelatin*]*_total_* is the initial concentration of gelatin (in films) and [*Gelatin*]*_supernatant_* is the final gelatin concentration in the medium at different time points.

### Electrophoretic Analysis

Protein patterns of gelatin released from the ADA-GEL films of different compositions after 7 days of incubation in serum free DMEM were analyzed using sodium dodecyl sulfate-polyacrylamide gel electrophoresis (SDS-PAGE). SDS-PAGE was carried out using the Mini-PROTEAN 3 Cell system (Bio-Rad, Germany). The resolving gels (10% acrylamide of about 1.0 mm thickness) were prepared according to the method described by Laemmli [39]. Supernatants collected after 7 days during the degradation study were heated at 90°C for 5 min and then analyzed by electrophoresis which was run at a constant voltage (120 V). Pre-stained molar mass markers were used as standards (Thermo Scientific, Germany). Proteins were visualized using silver staining.

### Cell Seeding and Cultivation

The primary cells, normal human dermal fibroblasts (NHDF) (Promocell, Germany), were cultured in DMEM supplemented with 10% (v/v) FCS and 1% (v/v) antibiotic-antimycotic, at 37°C, with a controlled atmosphere of 5% CO_2_ and 95% relative humidity. Monolayer of NHDF in their growth phase (∼90% confluence) was detached using trypsin/1 mM ethylenediaminetetraacetic (EDTA) (Life Tech., Germany) in PBS, centrifuged and resuspended in complete cell culture medium. Cells were counted using trypan blue exclusion method (Sigma-Aldrich, Germany) before seeding on hydrogels.

The prepared circular films of alginate and ADA-GEL hydrogels were placed in the wells of 24-well plates (VWR Int., Germany) and washed with DMEM. For analysis of cell adhesion, 85000 cells/film were seeded and incubated in a humidified atmosphere of 95% relative humidity and 5% CO_2_, at 37°C. Culture medium was changed on the next day after seeding, and then every two days.

### Mitochondrial Activity

Mitochondrial activity of seeded NHDF on different hydrogel films was assessed through the enzymatic conversion of tetrazolium salt (WST-8 assay kit, Sigma Aldrich, Germany) after 4 and 7 days of cultivation. Culture media were completely removed from the samples and freshly prepared culture medium was added containing 1 v% WST-8 assay kit, prior to the incubation for 2 hours. Subsequently, 100 µl of supernatant from each samples was transferred into a well of a 96 well-plate and measured the absorbance at 450 nm with a microplate reader (PHOmo, autobio labtec instruments co. Ltd. China).

### Cell Staining

To assess the viability of cells, live staining was performed with calcein acetoxymethyl ester (Calcein AM, Invitrogen, USA) after 4 and 7 days of cultivation, and nuclei were visualized by blue nucleic acid stain, DAPI (4',6-diamidino-2-phenylindole, dilactate, Invitrogen, USA) which preferentially bind to A (Adenine) and T (Thymine) regions of DNA. To investigate the cell morphology after 4 and 7 days of cultivation, cells were stained with rhodamine phalloidin (Invitrogen, USA), which selectively stains F-actin and nuclei were visualized with green nucleic acid stain, SYTOX (Invitrogen, USA). The images of calcein-DAPI and phalloidin-SYTOX stained cells were taken by fluorescence microscope (FM) (Axio Scope A.1, Carl Zeiss Microimaging GmbH, Germany).

### Cell Counting

Automatic cell counting in the fluorescence images, taken as described above, was performed using the ImageJ software (version 1.47v, National Institutes of Health, Bethesda, MD, USA). Six images of two different magnifications (10× and 20×) per samples were used to analyze the cell number by counting the nuclei of cells. The entire area of the image was calculated and the result was presented as number of cells per unit area of sample.

### Scanning Electron Microscopy (SEM) Analysis

In subsets of experiments, the cell morphology on hydrogel films after 4 and 7 days of cultivation was analyzed by SEM (LEO 435 VP, LEO Electron Microscopy Ltd, Cambridge, UK). Briefly, after 4 and 7 days of cultivation, NHDF seeded films were fixed and dehydrated in a graded ethanol series (30, 50, 70, 80, 90, 95, and 99.8 v%). Then the samples were critical-point dried with a critical point dryer (Leica EM CPD300, Germany) and coated with gold sputter before SEM examination.

### Statistics

Statistical analyses of mitochondrial activity of NHDF and cell numbers were accomplished by one-way analysis of variance (ANOVA) on the ADA-GEL hydrogels of different compositions compared to alginate after 4 and 7 days of incubation. The pairwise comparison of the means was performed with the Bonferroni's test (post hoc comparison). *p*-values<0.05 were considered statistically significant.

## Results and Discussion

### Molar Mass of Alginate and ADA

The molar mass of alginate and synthesized ADA was analyzed by the intrinsic viscosity method as shown in Table 2. The intrinsic viscosity of alginate and ADA was measured by plotting the reduced and inherent viscosities which are presented in Figure S1 (supplementary information). The molar mass of alginate decreased significantly after periodate oxidation, which depends on the periodate equivalent used for oxidation reaction [40]. In the present study, 30 mol% equivalent of periodate was used for oxidation of alginate. This result is attributed to the scission of polysaccharide chain of alginate during oxidation. The periodate oxidation preferentially cleaves the vicinal glycols in guluronate unit of alginate to form dialdehyde derivatives [23], [41].

**Table 2:** Intrinsic viscosity and calculated molar mass of alginate and ADA.

Materials	Intrinsic viscosity (dL/g)	Molar mass (kDa)
Alginate	8.45 ± 0.11	422.3 ± 5.3
ADA	3.71 ± 0.06	185.5 ± 2.8

### Water Uptake Behavior

**Figure 1:**
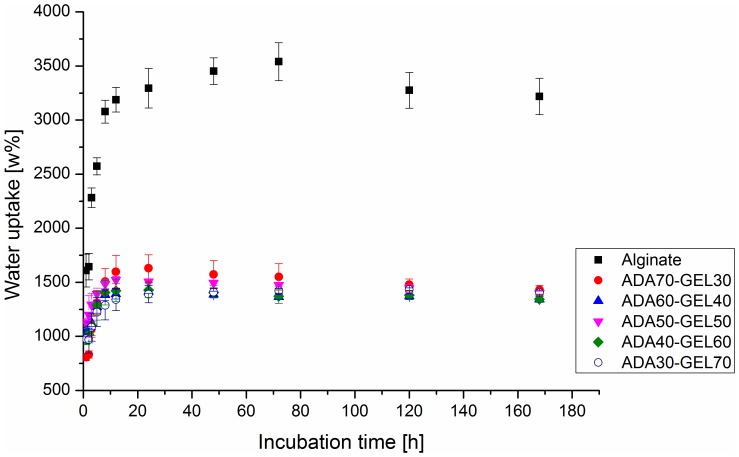
Water uptake behavior of alginate and ADA-GEL hydrogels. Water uptake as a function of incubation time in DMEM of the films fabricated from alginate and ADA-GEL hydrogels of different compositions.

Water uptake, one of the most important properties of hydrogel-based biomaterials, reflects the ability of the hydrogel to keep and diffuse water, which is related to its ability to absorb body fluid and transfer cell nutrients and metabolites [42], [43]. Water uptake of the films fabricated from alginate and ADA-GEL of different compositions was analyzed in cell culture medium at 37°C to understand the water uptake ability of hydrogels at conditions mimicking the *in vitro* study. As shown in Figure 1, water uptake of alginate and ADA-GEL films increased rapidly up to 12 hours. However, water uptake of alginate films was much higher than the water uptake of ADA-GEL films. After 72 hours of incubation, water uptake of alginate films was found to be 2.4 times greater than ADA50-GEL50 films. This effect was likely resulting from the fact that molar mass of ADA was significantly decreased due to the partial oxidation of alginate, which cleaves the vicinal glycols in alginate [20] and reduces the water uptaking ability of ADA-GEL. In addition, due to the crosslink of ADA and gelatin, the conformation of polysaccharide was changed which might further influence the water uptake properties. At longer incubation time points, no obvious difference was observed among the different compositions of ADA-GEL.

### *In Vitro* Swelling and Degradation Study

**Figure 2:**
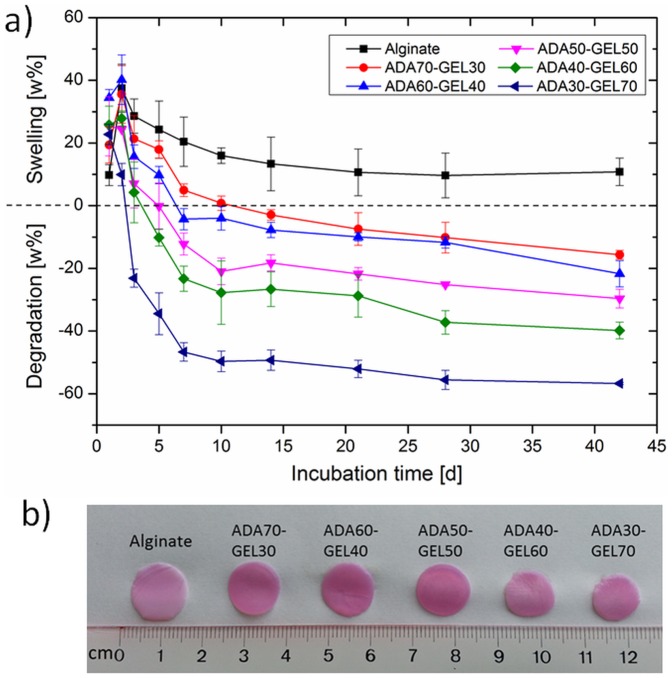
Swelling and degradation characteristics of alginate and ADA-GEL hydrogels. (a) Swelling and degradation as a function of incubation time in DMEM of the films fabricated from alginate and ADA-GEL hydrogels of different compositions and (b) photograph of the hydrogel films after 28 days of incubation during degradation study.

*In vitro* swelling and degradation of the hydrogel films of alginate and ADA-GEL were investigated by evaluating the weight gain and weight loss, respectively, of the hydrogel films during different incubation times in DMEM, at 37°C, with a controlled atmosphere of 5% CO_2_ and 95% relative humidity. As shown in Figure 2 (a), at the beginning of incubation, we observed the swelling of all hydrogels. However, within 1–2 days, the degradation of ADA-GEL hydrogels had started. ADA-GEL hydrogels underwent fast weight loss during the first 10 days. Moreover, ADA50-GEL50, ADA40-GEL60 and ADA30-GEL70 exhibited a considerably faster degradation profile as compared to the other two ADA-GEL hydrogels. Over longer incubation times, the weight loss of ADA-GEL hydrogels of all compositions was slowed down remarkably, reaching a plateau after approximately 14 days. After 42 days of incubation, the weight loss of ADA70-GEL30, ADA60-GEL40, ADA50-GEL50, ADA40-GEL60 and ADA30-GEL70 hydrogels were 15.6, 21.7, 29.6, 39.8 and 56.7%, respectively. Usually, an increase in crosslinking degree results in a decrease in the weight loss of a hydrogel [44], [45]. For ADA-GEL hydrogel, the higher content of ADA contributed to the formation of a highly crosslinking network because of the available aldehyde groups which act as sites for crosslinking with free ε-amino groups of gelatin. It must be noted that comparatively high amount of gelatin remained uncrosslinked in high gelatin-containing ADA-GEL hydrogels due to the smaller amount of available aldehyde groups. As a result, high amount of uncrosslinked gelatin leached out rapidly from high gelatin-containing ADA-GEL hydrogels, which contributed to the observed high rate of weight loss. Moreover, as shown above, ADA possesses low molar mass compared to alginate because of periodate oxidation during synthesis of ADA which cleaves the vicinal glycols of alginate. This phenomenon enhances the degradation property of ADA [24]. Thus, the degradation behavior of ADA-GEL hydrogels is strongly influenced by the degradation of both components, ADA and gelatin. In contrast, there was no obvious degradation found for alginate hydrogel. Moreover, alginate showed stronger swelling behavior compared to ADA-GEL hydrogels over the whole incubation period. Alginate and ADA-GEL hydrogel films after 28 days of incubation during degradation study are shown in Figure 2 (b). It is clearly visible that the size of the alginate film is larger than that of ADA-GEL films. Moreover, ADA-GEL films become smaller with increasing gelatin content which reflects the results obtained from the degradation study. These results proved that the biodegradability of ADA-GEL hydrogel is controllable by changing its composition. This feature is of critical importance for the application in tissue engineering, where the degradation kinetic of biomaterials should be matched to the tissue regeneration process [46], [47]. Moreover, the degradation behavior of alginate and ADA-GEL hydrogels was investigated by measuring the storage moduli of the hydrogels in our previous study [48]. Storage moduli of alginate and ADA50-GEL50 hydrogels over the same incubation period of the *in vitro* degradation study were analyzed by dynamic mechanical thermal analyzer (DMTA) over a frequency ranging from 0.1 to 15 Hz. The storage moduli of the hydrogels, alginate and ADA50-GEL50 were found to be of the same order of magnitude (from around 400 kPa at 0.1 Hz to around 600 kPa at 15 Hz) before incubation. The storage moduli of alginate and ADA50-GEL50 hydrogels decreased over the incubation period. However, the rate of loss of the storage moduli was higher for ADA50-GEL50 compared to alginate. After 28 days of incubation, the storage moduli of alginate and ADA50-GEL50 were found to be 196 and 113 kPa, respectively at 15 Hz, which proves the higher degradability of ADA50-GEL50 compared to alginate.

### Gelatin Release Behavior

**Figure 3:**
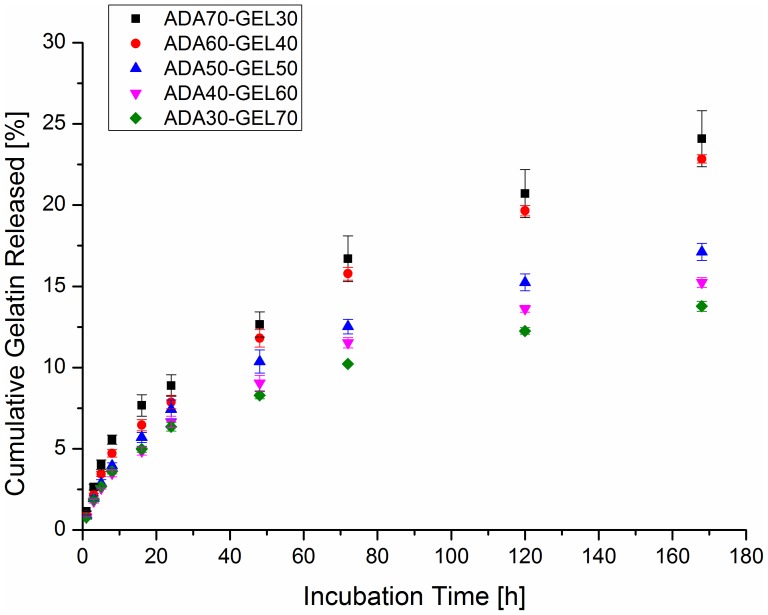
Gelatin release pattern from ADA-GEL hydrogels. Cumulative gelatin release as a function of incubation time in DMEM from the films fabricated from ADA-GEL hydrogels of different compositions.

The gelatin release from ADA-GEL films of different compositions was monitored in DMEM at 37°C to understand the release behavior of gelatin during cell culture studies. As shown in Figure 3, gelatin release from hydrogels (w%) was very low even after 7 days (168 h), which was expected, as gelatin was covalently crosslinked with ADA [49]. Nonetheless, the release of gelatin started immediately after immersion in DMEM, and gelatin release rate was slowed down upon prolonged incubation period. The amount of gelatin release was higher in high gelatin-containing ADA-GEL films. Although the *amount* of gelatin released from those films was higher, their released *percentage* was found to be lower. This phenomenon might be explained by an increased extent of physical interactions due to high gelatin content [48]. It has been reported that the renaturation of gelatin is totally intramolecular at low gelatin concentration, and becomes intermolecular at high concentration [50]. This phenomenon may slow down the released percentage of gelatin from high gelatin containing ADA-GEL films.

### Electrophoretic Analysis

**Figure 4:**
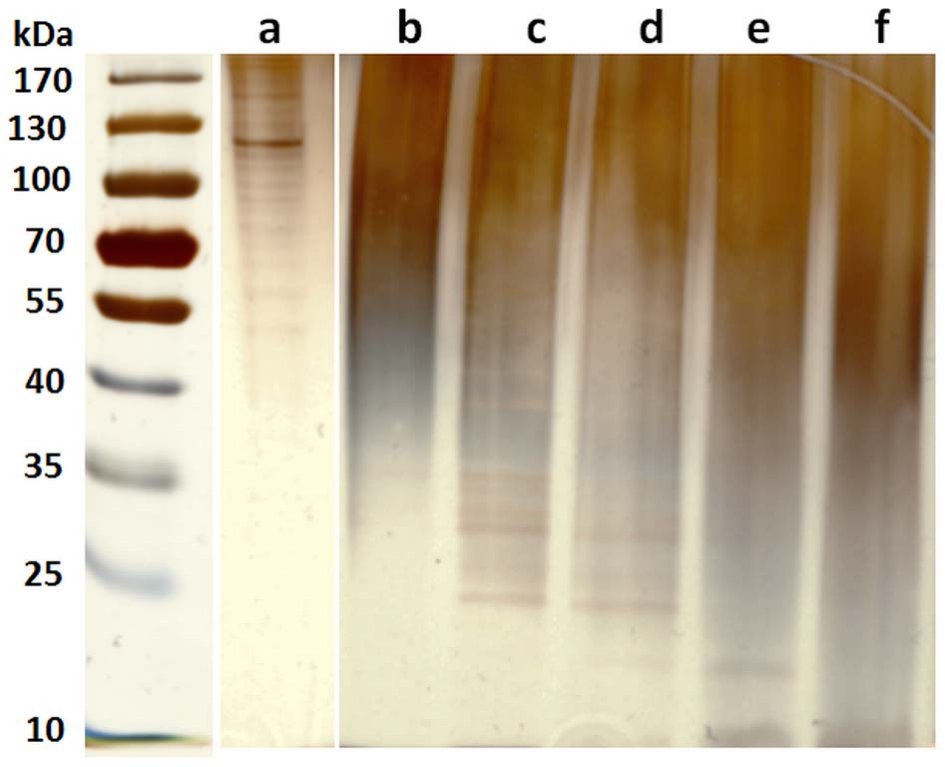
Electrophoretic analysis of protein in the released supernatant from ADA-GEL hydrogels. Silver stained SDS-PAGE gel indicating the protein patterns according to the molecular weight of (a) pure gelatin. After 7 days of incubation, the polypeptide patterns of gelatin released from (b) ADA70-GEL30, (c) ADA60-GEL40, (d) ADA50-GEL50, (e) ADA40-GEL60 and (f) ADA30-GEL70.

To analyze the molar mass of released proteins in the supernatant (DMEM) after 7 days of incubation, we performed SDS-PAGE study. In Figure 4, pure gelatin is seen to exhibit a wide molar mass distribution from 53 to 180 kDa. However, prominent bands appeared at the molar mass region ranged from 70 to 150 kDa which proves that gelatin consists of polypeptides of α-chains [51], [52]. Interestingly, after 7 days of incubation at 37°C, most of the bands were detected at a molar mass lower than 50 kDa for the ADA-GEL hydrogels. This result indicates that the gelatin molecules were fragmented to lower molar mass protein derivatives due to hydrolysis during the incubation period. Moreover, the molar mass of protein fragments decreased with increasing gelatin content in ADA-GEL hydrogels. Many narrow bands were observed at ∼25–40 kDa and ∼20–35 kDa for ADA60-GEL40 and ADA50-GEL50, respectively. However, there were no intermediate bands observed for ADA40-GEL60 and ADA30-GEL70. For these two compositions, the bands were observed only at very low molar mass (∼10–20 kDa). A possible explanation of this phenomenon is an enhanced hydrolytic degradation of gelatin of the ADA-GEL hydrogels with high gelatin content due to the high pH of the supernatant [53], [54]. Indeed, our results showed that the pH of supernatant after 7 days of incubation increased with increasing gelatin content in ADA-GEL hydrogels. The pH of ADA70-GEL30, ADA60-GEL40, ADA50-GEL50, ADA40-GEL60 and ADA30-GEL70 were found to be 7.34, 7.42, 7.44, 8.18 and 8.25, respectively after 7 days of incubation. However, no bands appeared for the hydrogel of composition ADA70-GEL30 probably due to the very low concentration of protein in the supernatant.

### Cell Viability and Mitochondrial Activity

**Figure 5:**
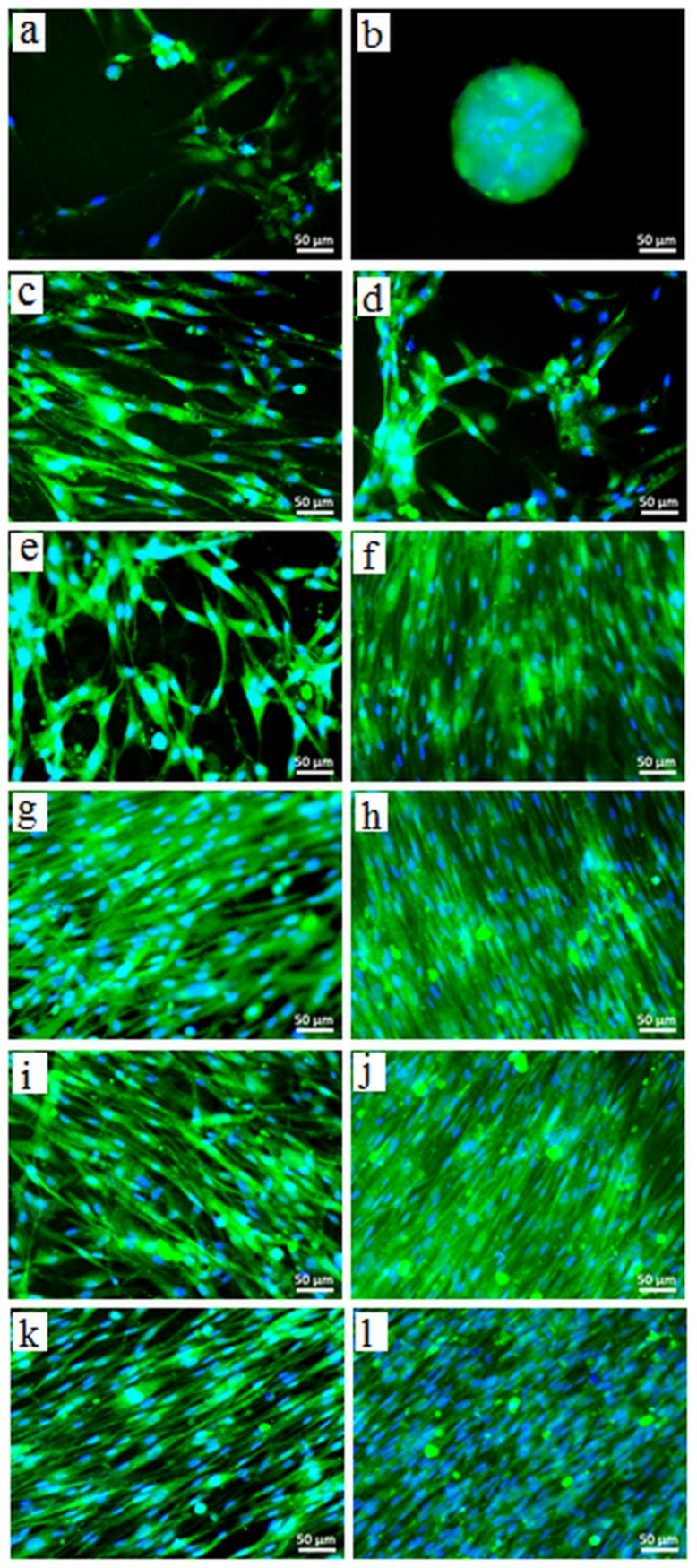
Assessment of NHDF viability and attachment on alginate and ADA-GEL hydrogels. FM images of NHDF adhered on (a,b) alginate, (c,d) ADA70-GEL30, (e,f) ADA60-GEL40, (g,h) ADA50-GEL50, (i,j) ADA40-GEL60, and (k,l) ADA30-GEL70 after 4 days (left column) and 7 days (right column) of incubation. The cells were stained for live cells (green) and nuclei (blue). Scale bar: 50 µm.

Figure 5 shows living NHDF (stained with Calcein AM) on alginate and ADA-GEL hydrogels after 4 and 7 days of incubation. The viability and morphology of living cells can be evaluated using calcein AM that stains the cytoplasm of living cells [55]. Esterases present in the cytoplasm of the living cells, break down calcein, resulting in a fluorescent green product which is impermeable to cell membranes. Apart from the cell viability calcein staining further gives information about the cell shape and membrane integrity which are the hallmarks of normal cell equilibrium. Cells were also stained with DAPI, a nucleic acid stain, in order to assess the integrity of the nucleus [56]. In Figure 5, the comparative reduction in viable cell numbers on alginate hydrogel after 4 days of incubation is shown. Interestingly, the cells were found to be agglomerated and formed clusters on alginate hydrogels after 7 days of incubation. This result indicates that on alginate hydrogel, cell-cell interactions are stronger than cell-material interactions, which results in a weakened attachment of cells to the material surface and the clustering of cells.

On ADA-GEL hydrogels, more viable cells with intact nuclei and cell membranes were found as compared with pure alginate. Moreover, cells were found to be attached and spread after 4 and 7 days of incubation. As expected, the number of adherent cells increased with increasing gelatin content in ADA-GEL hydrogels. This phenomenon can be ascribed to the availability of cell-binding peptides of gelatin in the hydrogel. ADA-GEL hydrogels of high gelatin content have more than required amount of gelatin for crosslinking with ADA. These hydrogels therefore possess more cell adhesion peptides which enhance their cell-material interaction. Moreover, it is clearly visible that the attachment and growth of NHDF occurs in one direction on high gelatin containing ADA-GEL hydrogels, whereas this type of orientation of NHDF is not observed for low gelatin containing ADA-GEL hydrogels.

**Figure 6:**
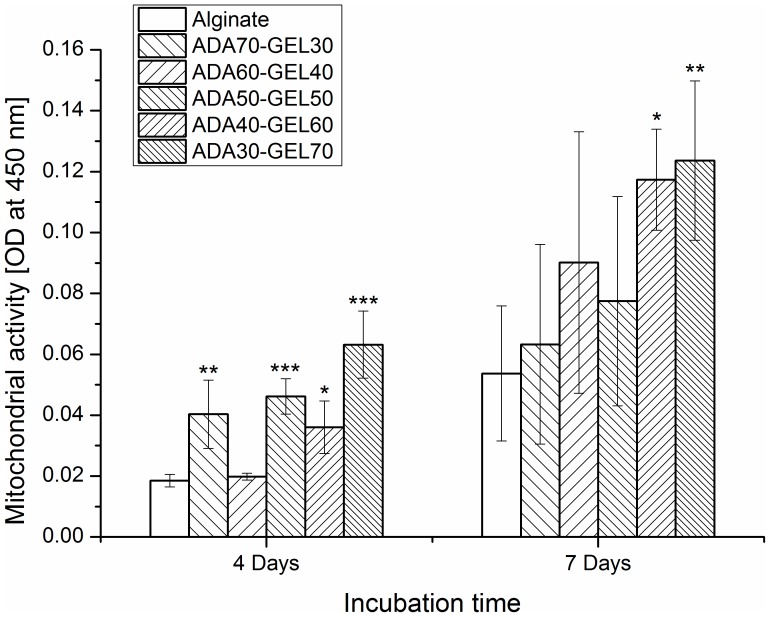
Evaluation of mitochondrial activity of NHDF on ADA-GEL hydrogels compared to alginate. Mitochondrial activity of NHDF on alginate and ADA-GEL hydrogels of different compositions after 4 and 7 days of cultivation. Alginate was used as the control material for each cultivation period. Asterisks denote significant difference compared with alginate films in each cultivation period, *p<0.05, **p<0.01 and ***p<0.001 (Bonferroni's post-hoc test was used).

Cell vitality plays an important role in living tissues, and it is therefore essential in tissue engineering and regeneration [57]. We found high number of viable cells growing on the ADA-GEL hydrogels, but their cell activity was not known. To answer this question, the metabolism of the NHDF on alginate and ADA-Gel hydrogels after 4 and 7 days of incubation was investigated by analyzing the mitochondrial activity of cells with WST-8 assay, as presented in Figure 6. In the metabolically active cells, tetrazolium compound is metabolized in the mitochondria and the product, formazan, is secreted into the medium. The resulting change in the medium color was measured spectrophotometrically and it reflects the relative mitochondrial activity of the cells. After 4 days of incubation, mitochondrial activity of NHDF grown on ADA-GEL hydrogels was significantly higher compared to those on alginate, except for ADA60-GEL40. After 7 days of incubation, mitochondrial activity of NHDF on ADA70-GEL30, ADA60-GEL40, ADA50-GEL50, ADA40-GEL60 and ADA30-GEL70 was 1.2, 1.7, 1.5, 2.2 and 2.3 folds increased as compared to that of alginate. However, these differences with alginate reached statistical significance only for high gelatin containing ADA-GEL hydrogels (ADA40-GEL60 and ADA30-GEL70). This result is in accordance with the results of the fluorescence staining of NHDF which are presented in Figure 5. Here, we also found a strong correlation between cell viability with the biodegradation of ADA-GEL hydrogels. Comparatively highly degradable ADA-GEL hydrogels (presented in Figure 2 (a) and 2 (b)) supported high cell viability. Degradation of hydrogel enhances the temporal changes of the bulk properties and helps the transport of matrix molecules which contributes to develop pericellular and extracellular matrices [47]. Moreover, due to degradation, the mesh size of the hydrogel increases which permits cell anchoring and penetration.

### Cell Number

**Figure 7:**
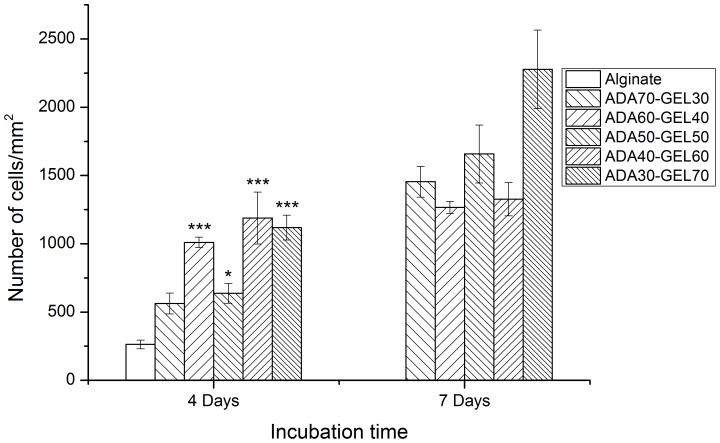
Quantification of cell number on ADA-GEL hydrogels compared to alginate by image analysis. Number of NHDF on alginate and ADA-GEL hydrogels of different compositions after 4 days and 7 days of cultivation. Alginate was used as the control material. Asterisks denote significant difference compared with alginate films in each cultivation period, *p<0.05, **p<0.01 and ***p<0.001 (Bonferroni's post-hoc test was used). Cells could not be counted due to the agglomeration of cells on alginate hydrogel after 7 days of cultivation.

**Figure 8:**
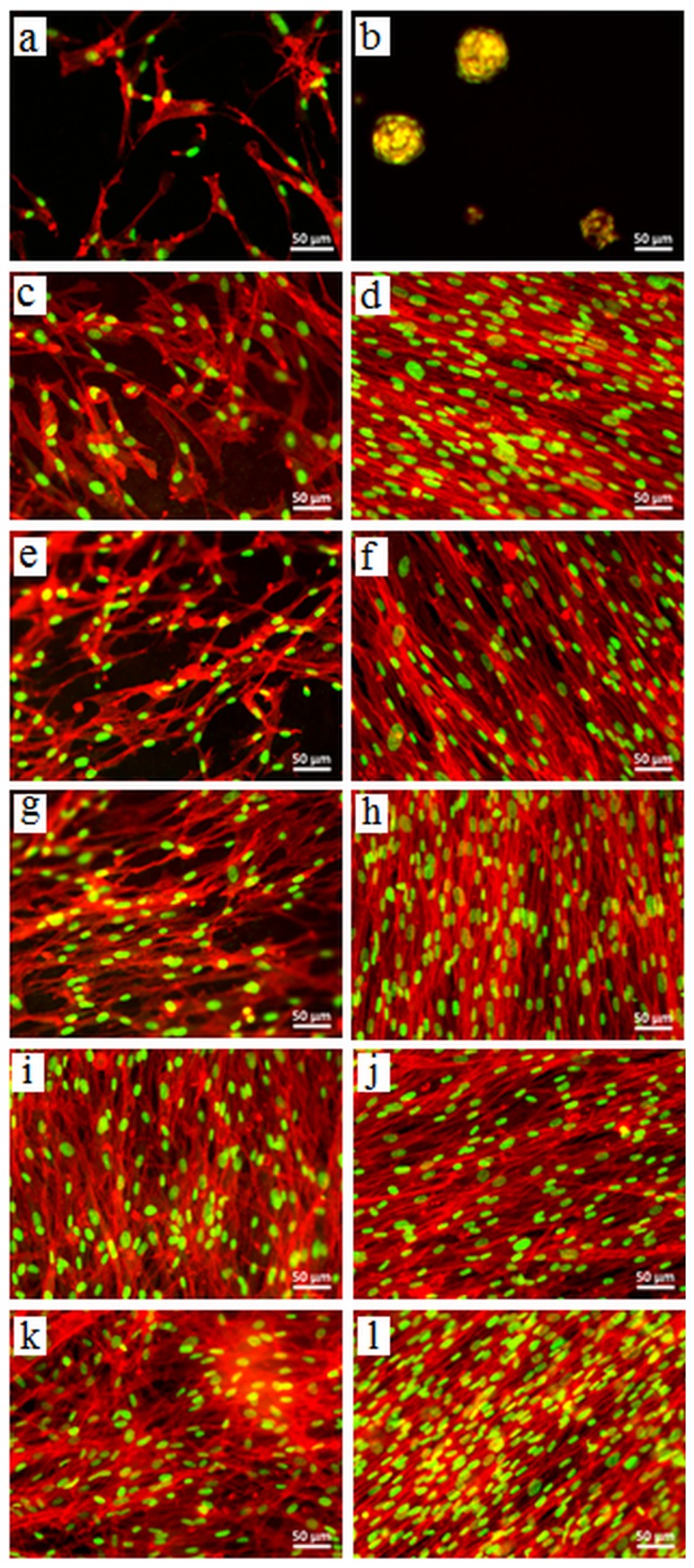
Morphological assessment of NHDF on hydrogels by analyzing F-actin pattern. FM images of NHDF adhered on (a,b) alginate, (c,d) ADA70-GEL30, (e,f) ADA60-GEL40, (g,h) ADA50-GEL50, (i,j) ADA40-GEL60, and (k,l) ADA30-GEL70 after 4 days (left column) and 7 days (right column) of incubation. The cells were stained for F-actin (red) and nuclei (green). Scale bar: 50 µm.

Number of cell was quantified by counting fluorescently stained cell nuclei with Image J software after specific cultivation periods. After 4 days of cultivation (Figure 7), the number of cells on ADA-GEL was significantly increased compared to alginate, except for ADA70-GEL30. After 7 days of cultivation, the number of cells for alginate is not shown in the Figure 7, because we could not count the cells on alginate hydrogel due to aggregation, which is shown in the fluorescence images of Figure 5 and 8. However, the cell number increased for all compositions of ADA-GEL hydrogel after 7 days of cultivation compared to that for 4 days of cultivation. Moreover, the cell number increased significantly (1.5, 1.8, 1.4 and 1.7 fold) for the highest gelatin containing ADA-GEL compared to ADA70-GEL30, ADA60-GEL40, ADA50-GEL50 and ADA40-GEL60, respectively, after 7 days of cultivation.

### Cell Morphology

To visualize cell morphology and cell spreading onto the hydrogels, actin cytoskeleton staining of the cells was performed using rhodamine phalloidin. Cell morphology plays an important role in the general cell hemostasis, and can be an early indicator of apoptotic responsiveness [58], or growth arrest [55]. The F-actin cytoskeleton staining showed that cells were having typical fibroblast morphology and, with increase in time and gelatin percentage in the hydrogels, an increase in cell number was observed, as shown in Figure 8. As expected, on the alginate hydrogel, there were markedly fewer cells as compared to the ADA-GEL hydrogels at day 4. Moreover, cellular morphology was disturbed and cell shape was rounded by day 7 (figure 8), which is in accordance with the results observed by calcein staining. On other gels, cells exhibited the normal fibroblasts morphology with increased number of cells which were spread onto the hydrogels on day 7. Fibroblasts generally develop a bipolar morphology with pseudopodial processes and always orient themselves nearly parallel to each other and to the substrate [59], which was clearly observed on ADA-GEL hydrogels by longer cultivation period (day 7). Moreover, both, the number of cells and the stress fibers increased with increasing gelatin content in ADA-GEL hydrogels.

**Figure 9:**
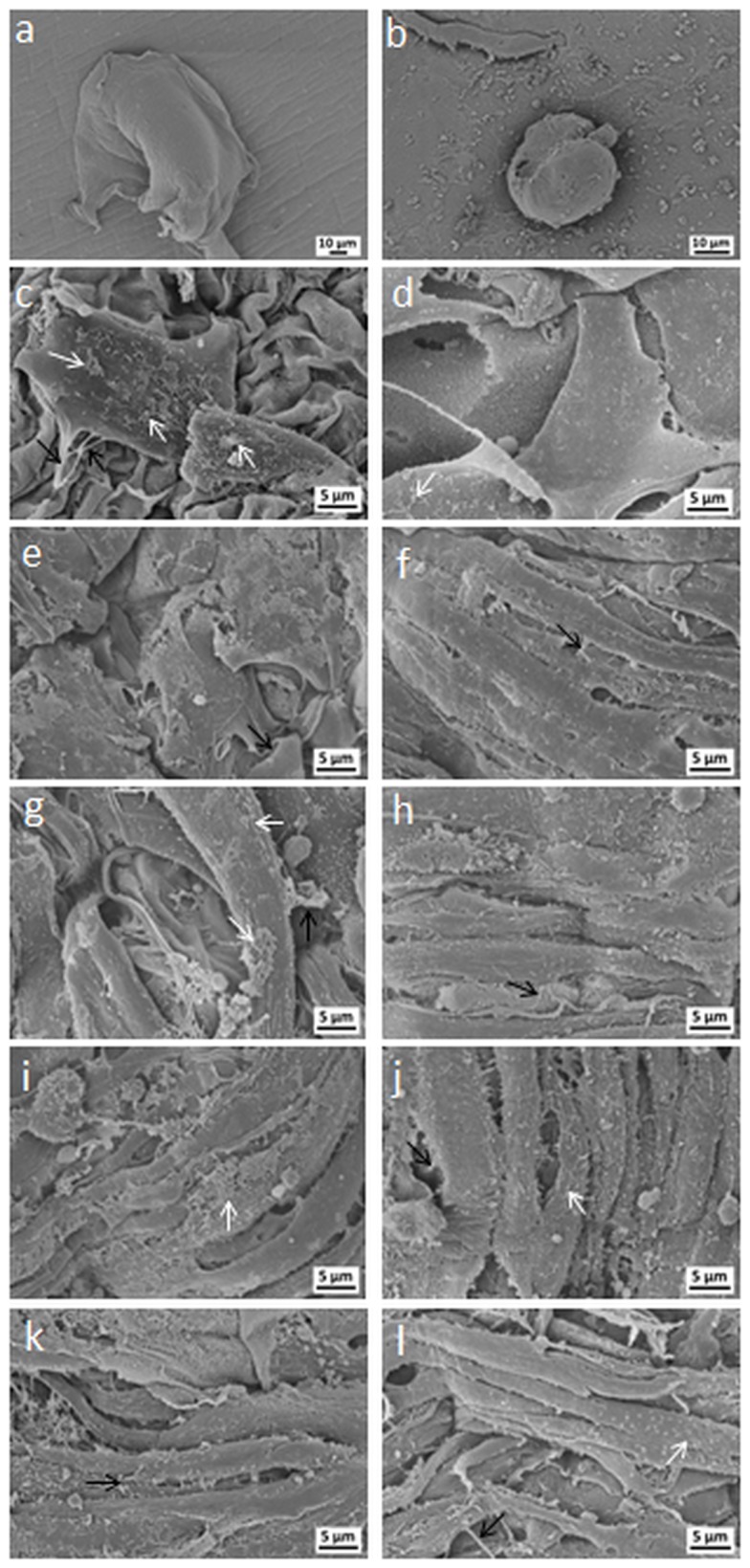
Morphological assessment of NHDF on hydrogels with SEM. SEM images of NHDF adherent on (a,b) alginate, (c,d) ADA70-GEL30, (e,f) ADA60-GEL40, (g,h) ADA50-GEL50, (i,j) ADA40-GEL60, and (k,l) ADA30-GEL70 after 4 days (left column) and 7 days (right column) of incubation. Black arrows and white arrows indicate filopodia and microvilli of cells, respectively.

To determine if there are morphological differences in the ways cells attach to the alginate and ADA-GEL hydrogels of different compositions, the nature of the cell-cell and cell-material interactions was investigated by SEM. Representative images are presented in Figure 9. As also observed by fluorescence staining, SEM images showed that cells did not adhere to alginate hydrogel and exhibited a round shape. On ADA-GEL hydrogels, the cells were firmly attached and the number of attached cells was growing with increasing content of gelatin in the hydrogels.

It is remarkable that the morphology of attached NHDF was found to be different on different compositions of ADA-GEL hydrogels. Cells were almost flat on ADA70-GEL30. However, the attached cells displayed typical elongated and spindle-like morphology on the ADA-GEL hydrogels with comparatively high gelatin content. Furthermore, after 7 days of cultivation, we observed that the cells covered the total surface of all ADA-GEL hydrogels, except for ADA70-GEL30. Moreover, the total surface of the high gelatin-containing ADA-GEL hydrogels (ADA40-GEL60 and ADA30-GEL70) was completely covered by cells already after 4 days of cultivation. With increasing cultivation period or gelatin content fibroblasts showed a preference for aligning in parallel to one another, which is an inherent population property of fibroblasts [60], [61]. However, non-parallel orientation of fibroblasts on ADA30-GEL70 hydrogel was observed after 7 days of cultivation. This particular lack of organization of fibroblasts was likely due to multilayering of cells on the hydrogel, in which sheets of cells grow over and across one another to produce superimposed layers of differently oriented cells [61]. These findings indicate that multiple layers of cells were formed on ADA30-GEL70 hydrogel, which result from intense matrix production by fibroblasts in these conditions. Moreover, it was observed that cells were anchored on the surface of ADA-GEL hydrogels of different compositions with their growing filopodia, which are indicated by black arrows in Figure 9. Many microvilli (marked by white arrows) were furthermore observed on the spread cells on ADA-GEL hydrogels, which represent a sign of highly active cells. It should be finally highlighted that microcapsules from ADA-GEL hydrogels have been fabricated as reported recently [23] and, in a parallel study, encapsulation of MG-63 osteosarcoma cells within ADA-GEL hydrogel has been shown [48]. In agreement with present results, it was observed that ADA-GEL hydrogel supported better cell proliferation, and led to higher mitochondrial and metabolic activities of encapsulated cells compared to alginate.

## Conclusions

Attachment, proliferation, spreading and viability of human dermal fibroblasts were significantly enhanced on ADA-GEL hydrogels synthesized by covalent crosslinking of ADA and gelatin. By this modification, we could successfully overcome the two critical drawbacks of alginate: its relatively poor cell adhesion properties as well as its relatively uncontrolled degradation characteristic. We observed that ADA-GEL hydrogels are highly biodegradable compared to alginate, which is an important factor in tissue regeneration. Moreover, ADA-GEL hydrogels with high gelatin content exhibited comparatively higher degradation and they better supported fibroblasts attachment, proliferation, spreading and viability. As the degradation kinetic of a biomaterial should correlate with the rate of tissue regeneration, our results prove that ADA-GEL hydrogels are promising materials for the application in tissue-engineering and regeneration.

## Supporting Information

Figure S1 **Calculation of intrinsic viscosity of alginate and ADA by plotting their reduced and inherent viscosities.** Reduced and inherent viscosities for (a) alginate and (b) ADA of various concentrations.
(TIF)
