# Supplementary figures and images for: Evaluation of Fibroblasts Adhesion and Proliferation on Alginate-Gelatin Crosslinked Hydrogel
Source: PLoS One. 2014 Sep 30;9(9):e107952. doi: 10.1371/journal.pone.0107952 (PMC4182442; doi:10.1371/journal.pone.0107952)

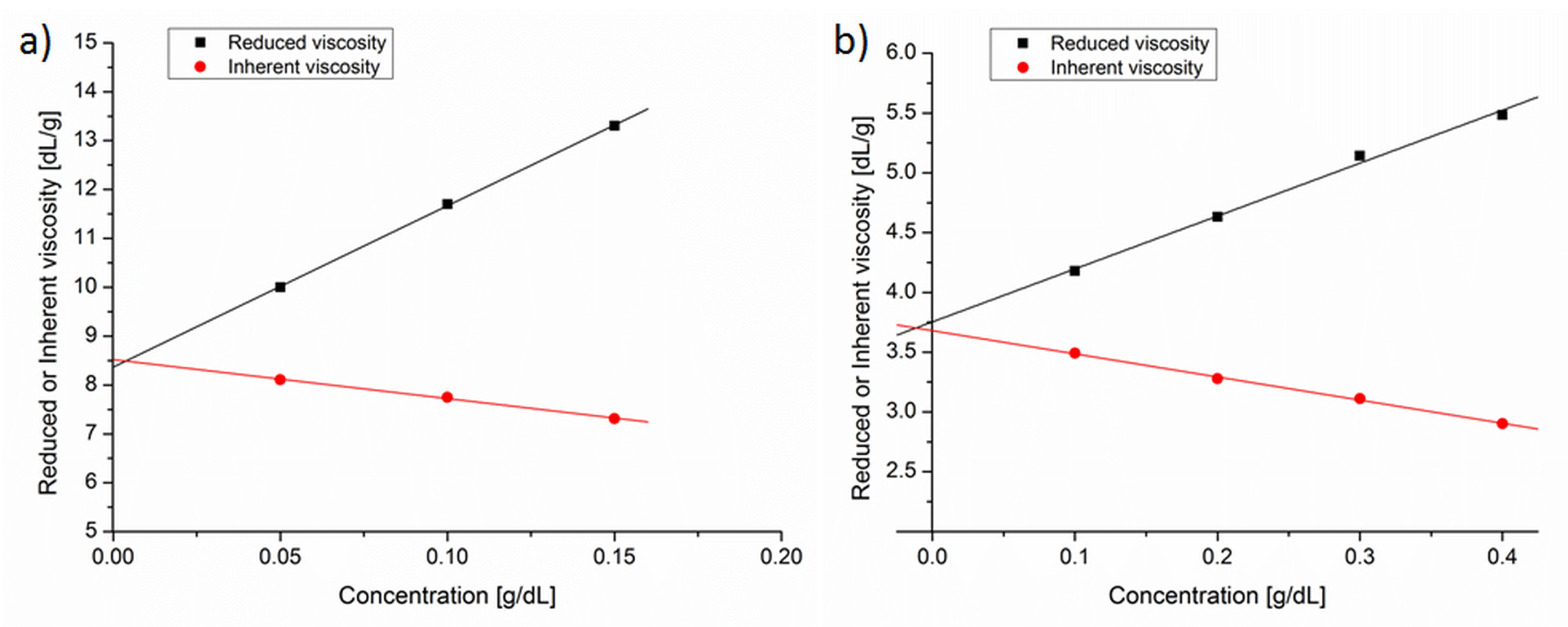

Supplement: Figure S1 — Calculation of intrinsic viscosity of alginate and ADA by plotting their reduced and inherent viscosities. Reduced and inherent viscosities for (a) alginate and (b) ADA of various concentrations. (TIF) [file pone.0107952.s001.tif]
